# Supplementary material for: Optimization of Oxford Nanopore Technology Sequencing Workflow for Detection of Amplicons in Real Time Using ONT-DART Tool
Source: Genes (Basel). 2022 Oct 3;13(10):1785. doi: 10.3390/genes13101785 (PMC9602318; doi:10.3390/genes13101785)
Supplement: Supplementary file 1 [file genes-13-01785-s001.zip › Player_et_al_2022_Genes_Supplemental_figures_S1-S12_abstract_graphic.pptx]

## Slide 1
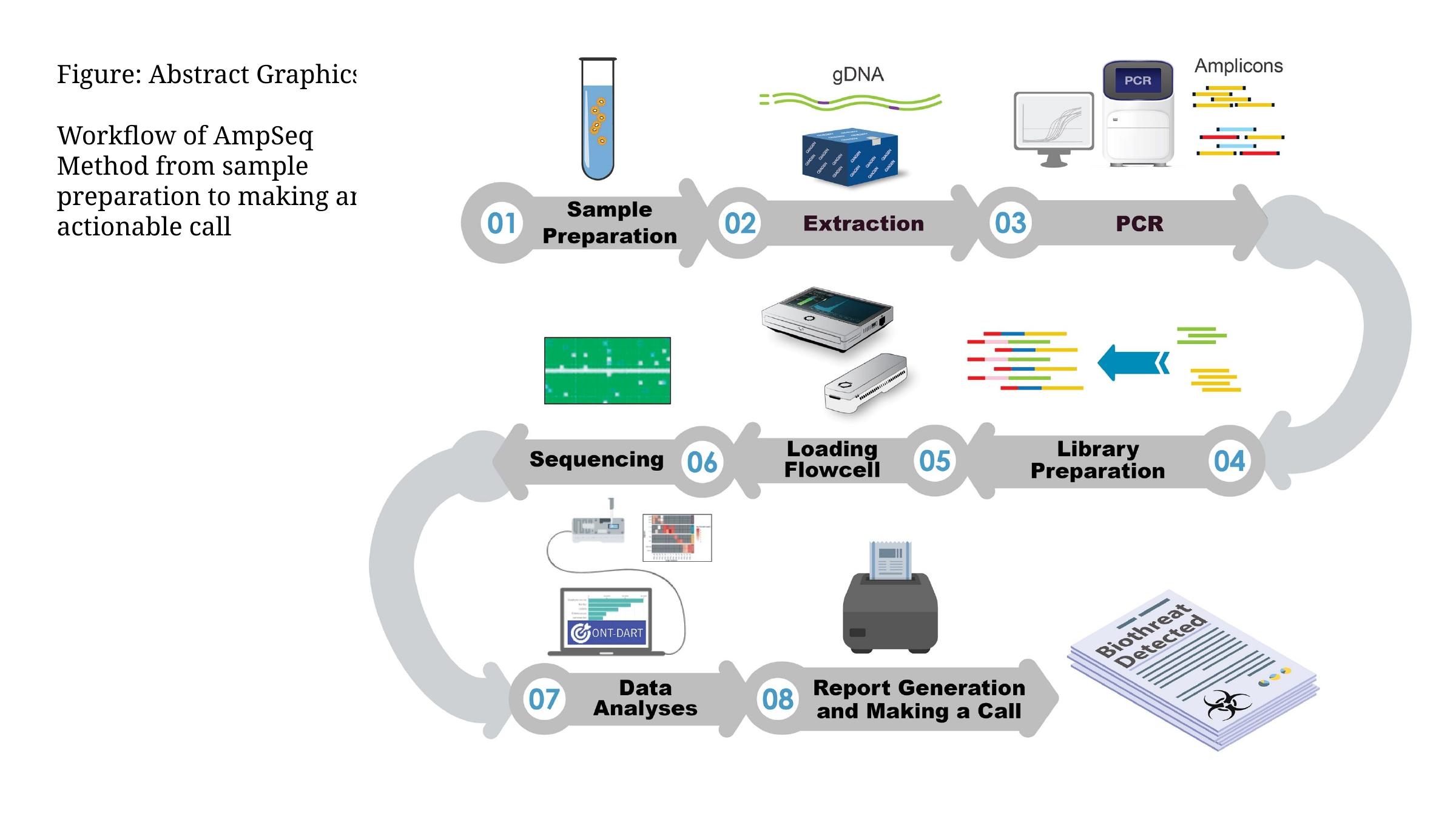

Figure: Abstract Graphics-
Workflow of AmpSeq Method from sample preparation to making an actionable call

## Slide 2
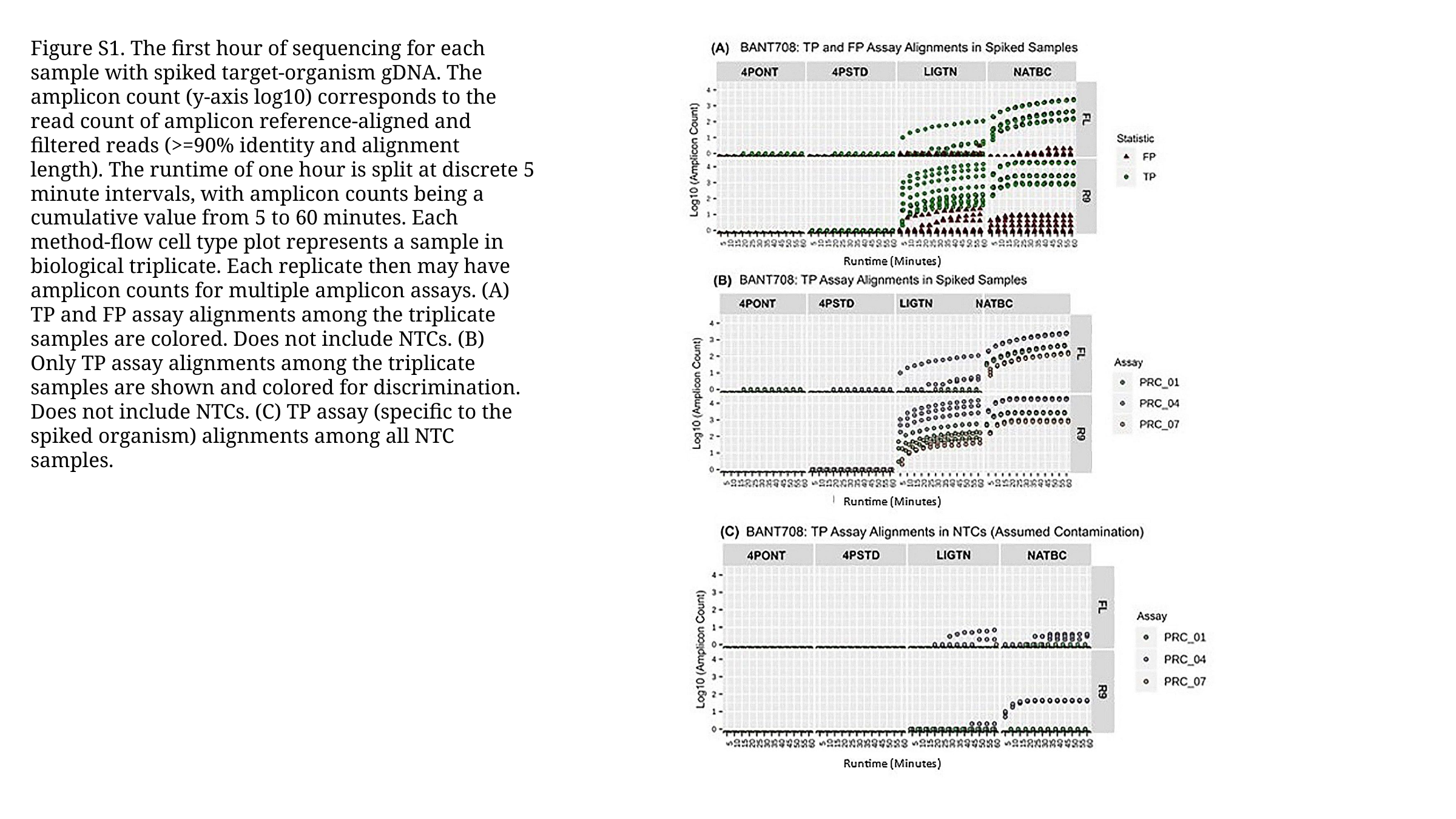

Figure S1. The first hour of sequencing for each sample with spiked target-organism gDNA. The amplicon count (y-axis log10) corresponds to the read count of amplicon reference-aligned and filtered reads (>=90% identity and alignment length). The runtime of one hour is split at discrete 5 minute intervals, with amplicon counts being a cumulative value from 5 to 60 minutes. Each method-flow cell type plot represents a sample in biological triplicate. Each replicate then may have amplicon counts for multiple amplicon assays. (A) TP and FP assay alignments among the triplicate samples are colored. Does not include NTCs. (B) Only TP assay alignments among the triplicate samples are shown and colored for discrimination. Does not include NTCs. (C) TP assay (specific to the spiked organism) alignments among all NTC samples.

## Slide 3
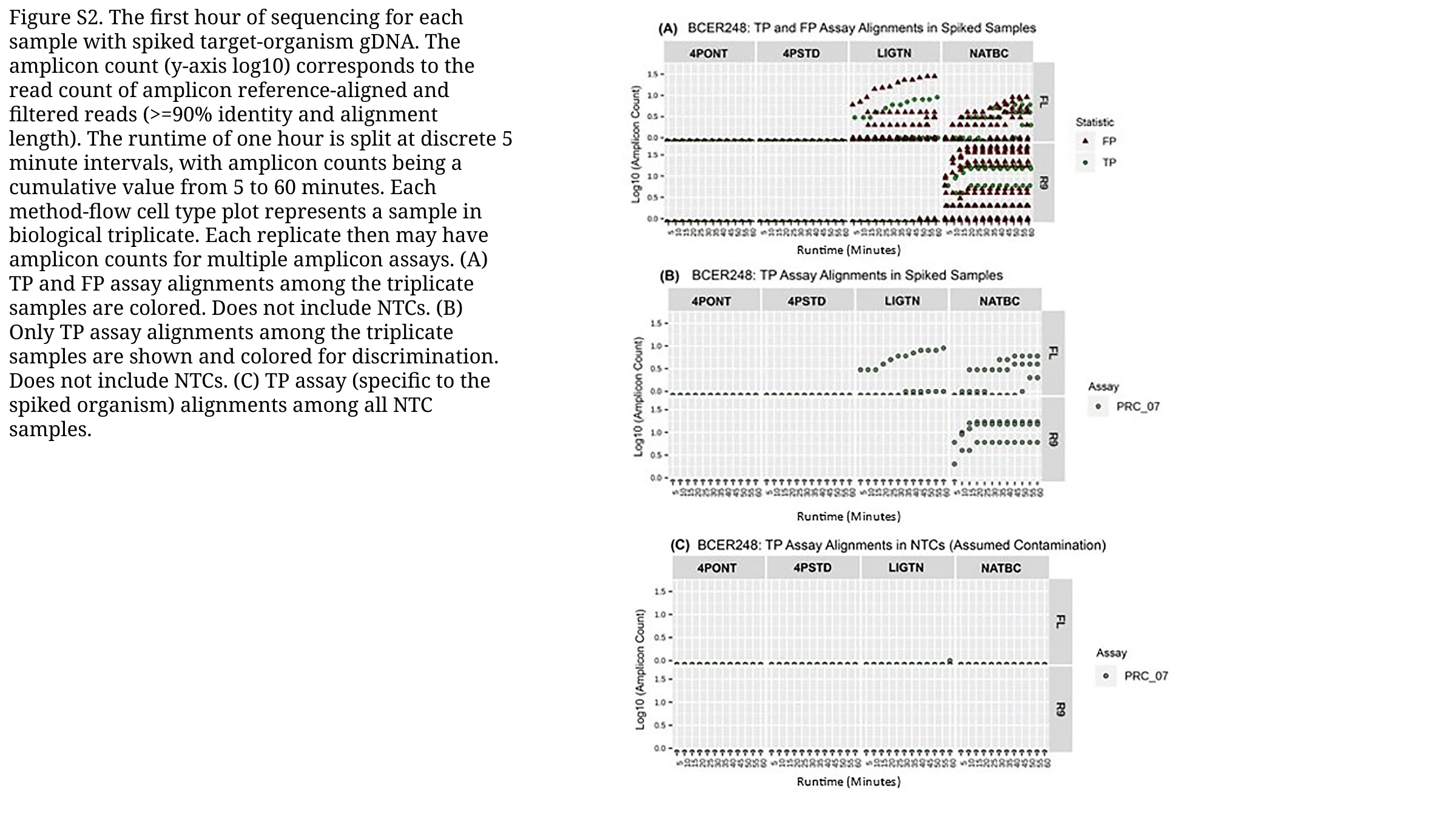

Figure S2. The first hour of sequencing for each sample with spiked target-organism gDNA. The amplicon count (y-axis log10) corresponds to the read count of amplicon reference-aligned and filtered reads (>=90% identity and alignment length). The runtime of one hour is split at discrete 5 minute intervals, with amplicon counts being a cumulative value from 5 to 60 minutes. Each method-flow cell type plot represents a sample in biological triplicate. Each replicate then may have amplicon counts for multiple amplicon assays. (A) TP and FP assay alignments among the triplicate samples are colored. Does not include NTCs. (B) Only TP assay alignments among the triplicate samples are shown and colored for discrimination. Does not include NTCs. (C) TP assay (specific to the spiked organism) alignments among all NTC samples.

## Slide 4
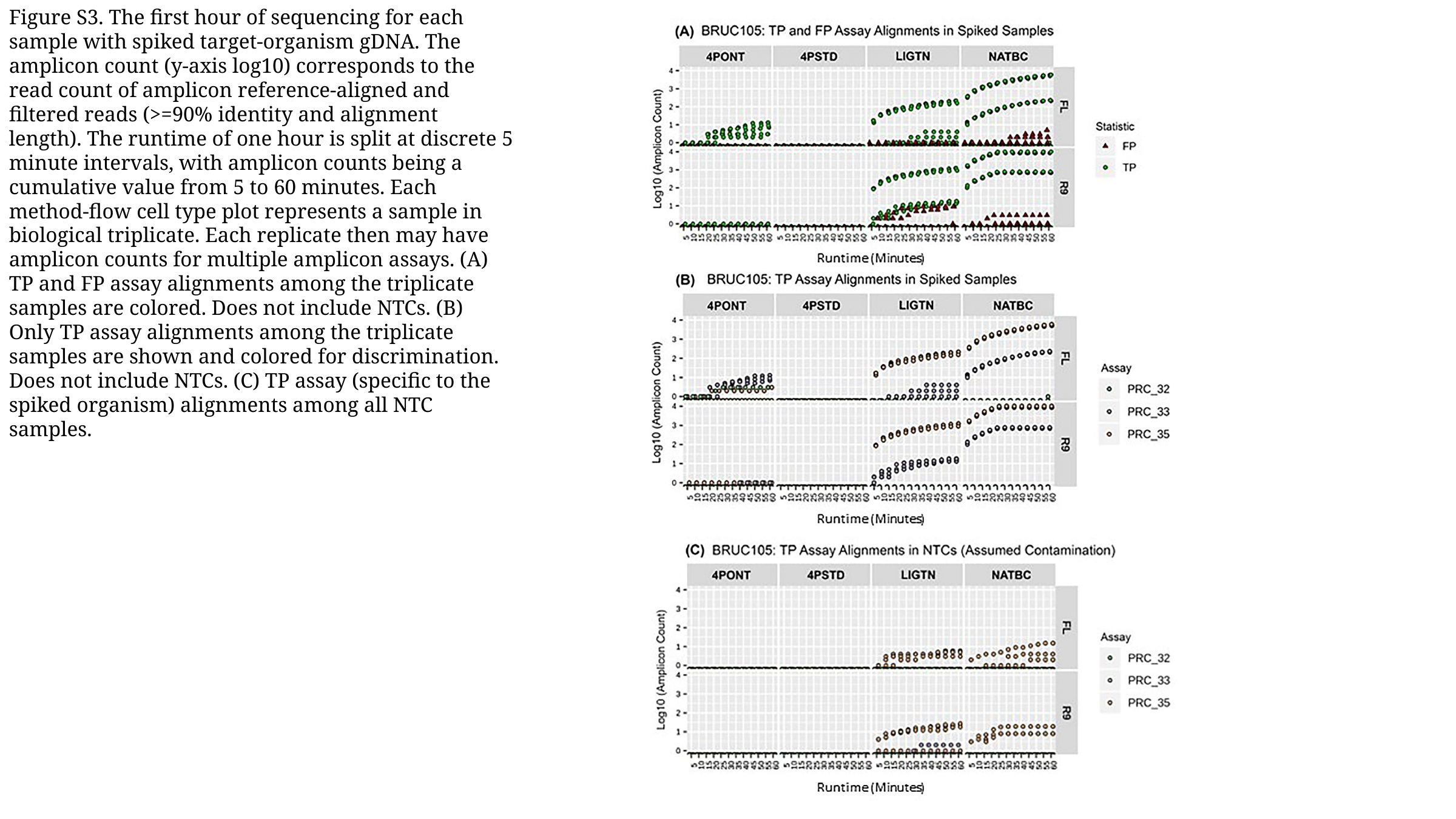

Figure S3. The first hour of sequencing for each sample with spiked target-organism gDNA. The amplicon count (y-axis log10) corresponds to the read count of amplicon reference-aligned and filtered reads (>=90% identity and alignment length). The runtime of one hour is split at discrete 5 minute intervals, with amplicon counts being a cumulative value from 5 to 60 minutes. Each method-flow cell type plot represents a sample in biological triplicate. Each replicate then may have amplicon counts for multiple amplicon assays. (A) TP and FP assay alignments among the triplicate samples are colored. Does not include NTCs. (B) Only TP assay alignments among the triplicate samples are shown and colored for discrimination. Does not include NTCs. (C) TP assay (specific to the spiked organism) alignments among all NTC samples.

## Slide 5
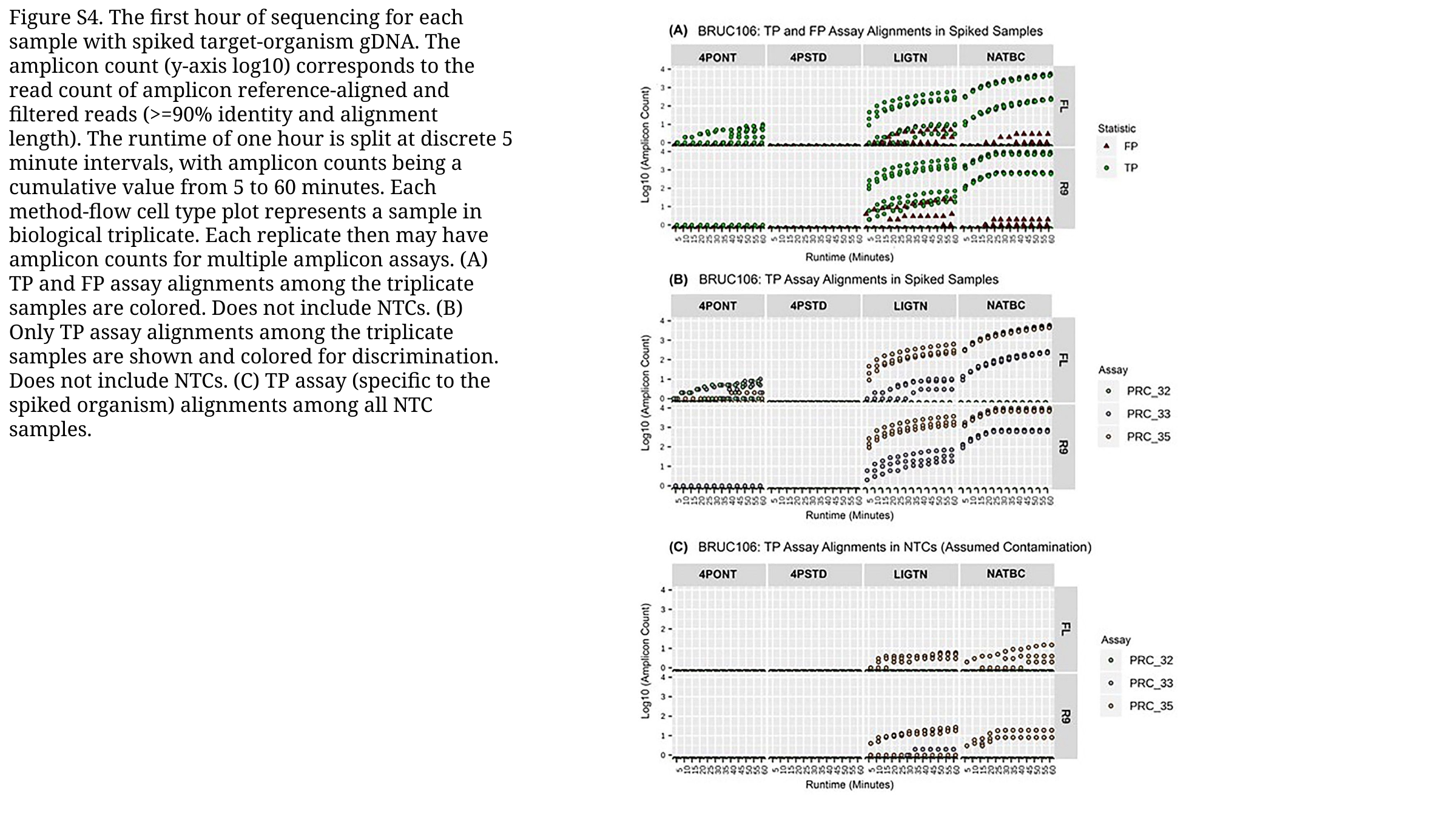

Figure S4. The first hour of sequencing for each sample with spiked target-organism gDNA. The amplicon count (y-axis log10) corresponds to the read count of amplicon reference-aligned and filtered reads (>=90% identity and alignment length). The runtime of one hour is split at discrete 5 minute intervals, with amplicon counts being a cumulative value from 5 to 60 minutes. Each method-flow cell type plot represents a sample in biological triplicate. Each replicate then may have amplicon counts for multiple amplicon assays. (A) TP and FP assay alignments among the triplicate samples are colored. Does not include NTCs. (B) Only TP assay alignments among the triplicate samples are shown and colored for discrimination. Does not include NTCs. (C) TP assay (specific to the spiked organism) alignments among all NTC samples.

## Slide 6
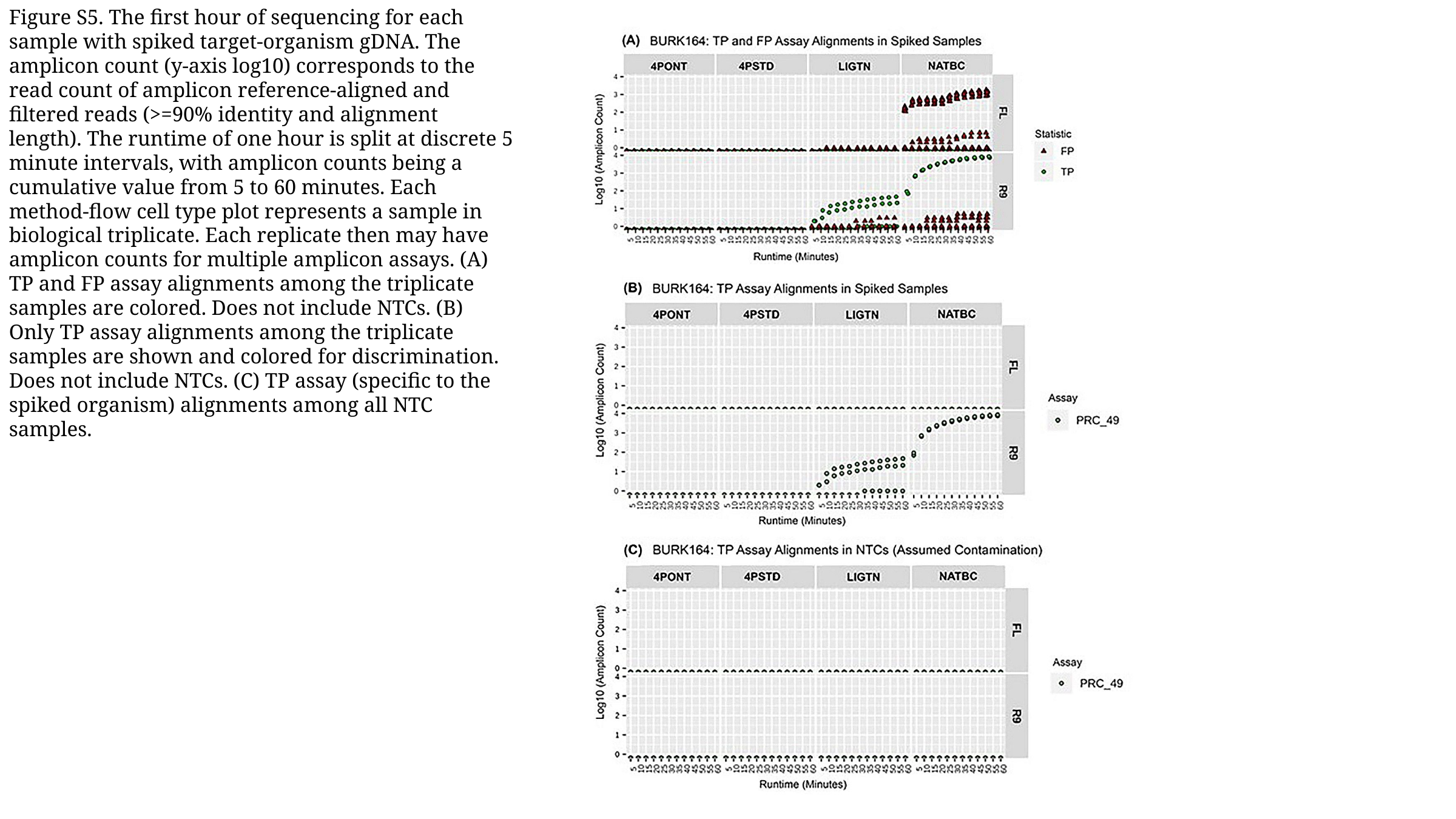

Figure S5. The first hour of sequencing for each sample with spiked target-organism gDNA. The amplicon count (y-axis log10) corresponds to the read count of amplicon reference-aligned and filtered reads (>=90% identity and alignment length). The runtime of one hour is split at discrete 5 minute intervals, with amplicon counts being a cumulative value from 5 to 60 minutes. Each method-flow cell type plot represents a sample in biological triplicate. Each replicate then may have amplicon counts for multiple amplicon assays. (A) TP and FP assay alignments among the triplicate samples are colored. Does not include NTCs. (B) Only TP assay alignments among the triplicate samples are shown and colored for discrimination. Does not include NTCs. (C) TP assay (specific to the spiked organism) alignments among all NTC samples.

## Slide 7
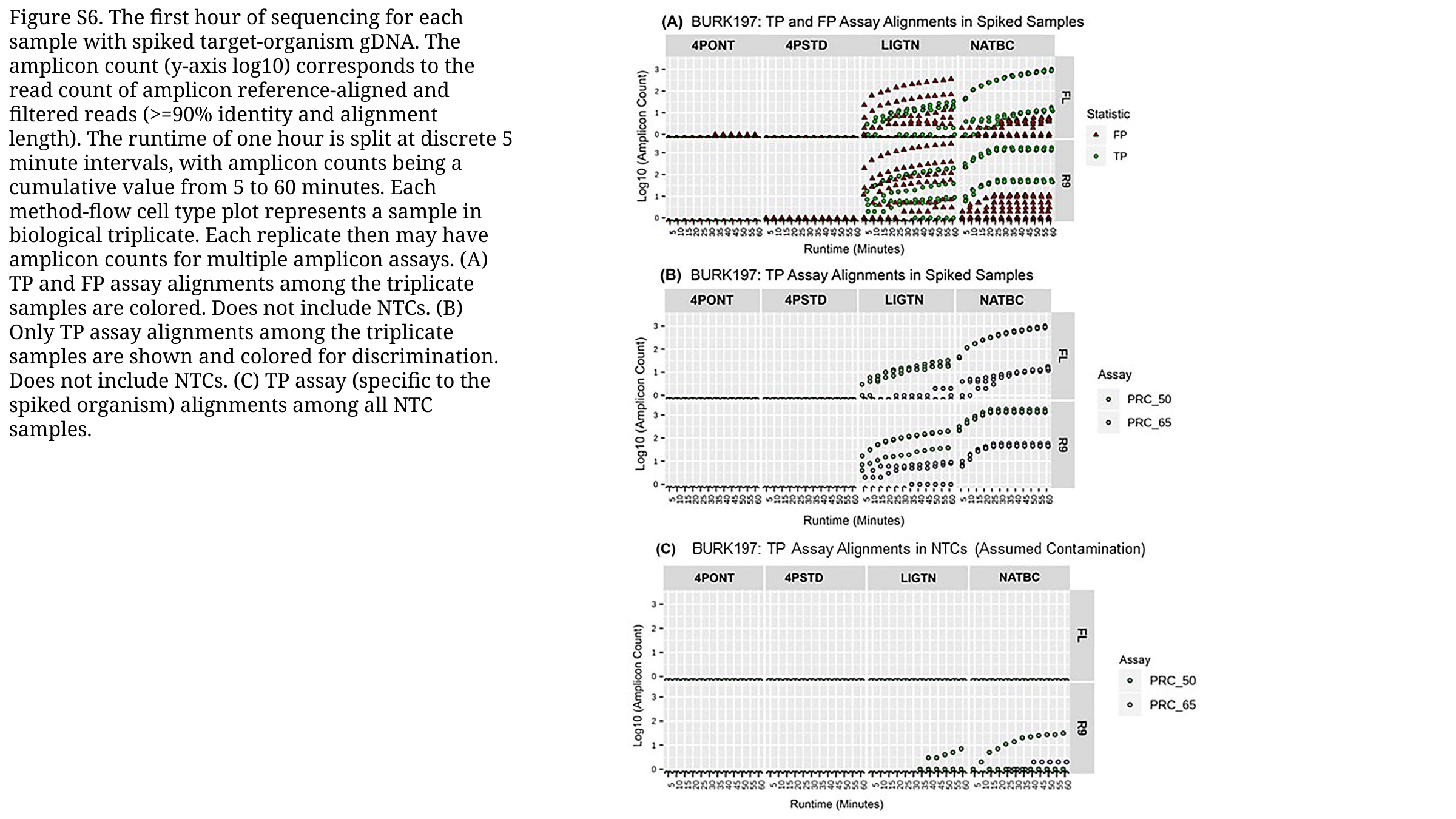

Figure S6. The first hour of sequencing for each sample with spiked target-organism gDNA. The amplicon count (y-axis log10) corresponds to the read count of amplicon reference-aligned and filtered reads (>=90% identity and alignment length). The runtime of one hour is split at discrete 5 minute intervals, with amplicon counts being a cumulative value from 5 to 60 minutes. Each method-flow cell type plot represents a sample in biological triplicate. Each replicate then may have amplicon counts for multiple amplicon assays. (A) TP and FP assay alignments among the triplicate samples are colored. Does not include NTCs. (B) Only TP assay alignments among the triplicate samples are shown and colored for discrimination. Does not include NTCs. (C) TP assay (specific to the spiked organism) alignments among all NTC samples.

## Slide 8
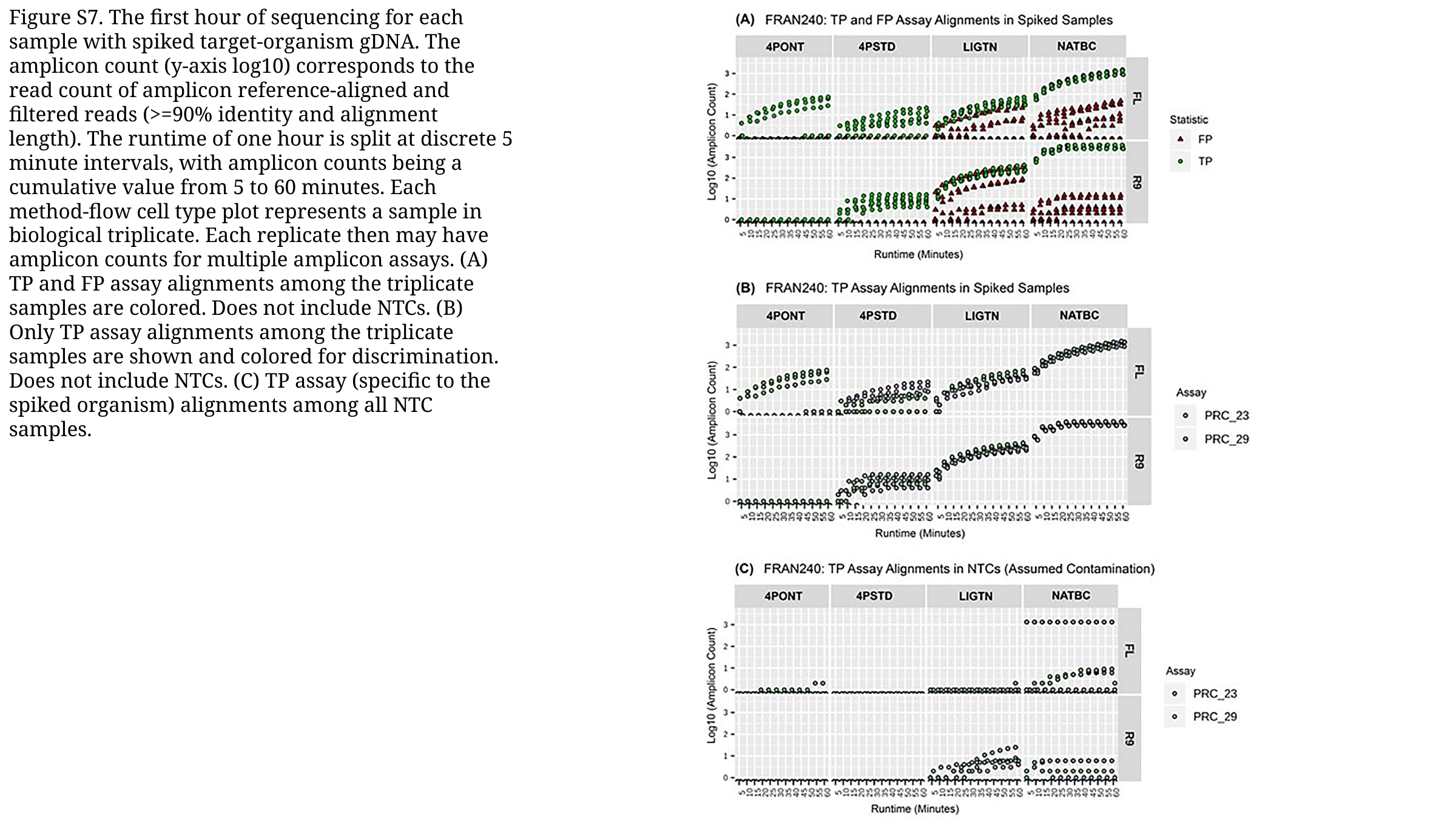

Figure S7. The first hour of sequencing for each sample with spiked target-organism gDNA. The amplicon count (y-axis log10) corresponds to the read count of amplicon reference-aligned and filtered reads (>=90% identity and alignment length). The runtime of one hour is split at discrete 5 minute intervals, with amplicon counts being a cumulative value from 5 to 60 minutes. Each method-flow cell type plot represents a sample in biological triplicate. Each replicate then may have amplicon counts for multiple amplicon assays. (A) TP and FP assay alignments among the triplicate samples are colored. Does not include NTCs. (B) Only TP assay alignments among the triplicate samples are shown and colored for discrimination. Does not include NTCs. (C) TP assay (specific to the spiked organism) alignments among all NTC samples.

## Slide 9
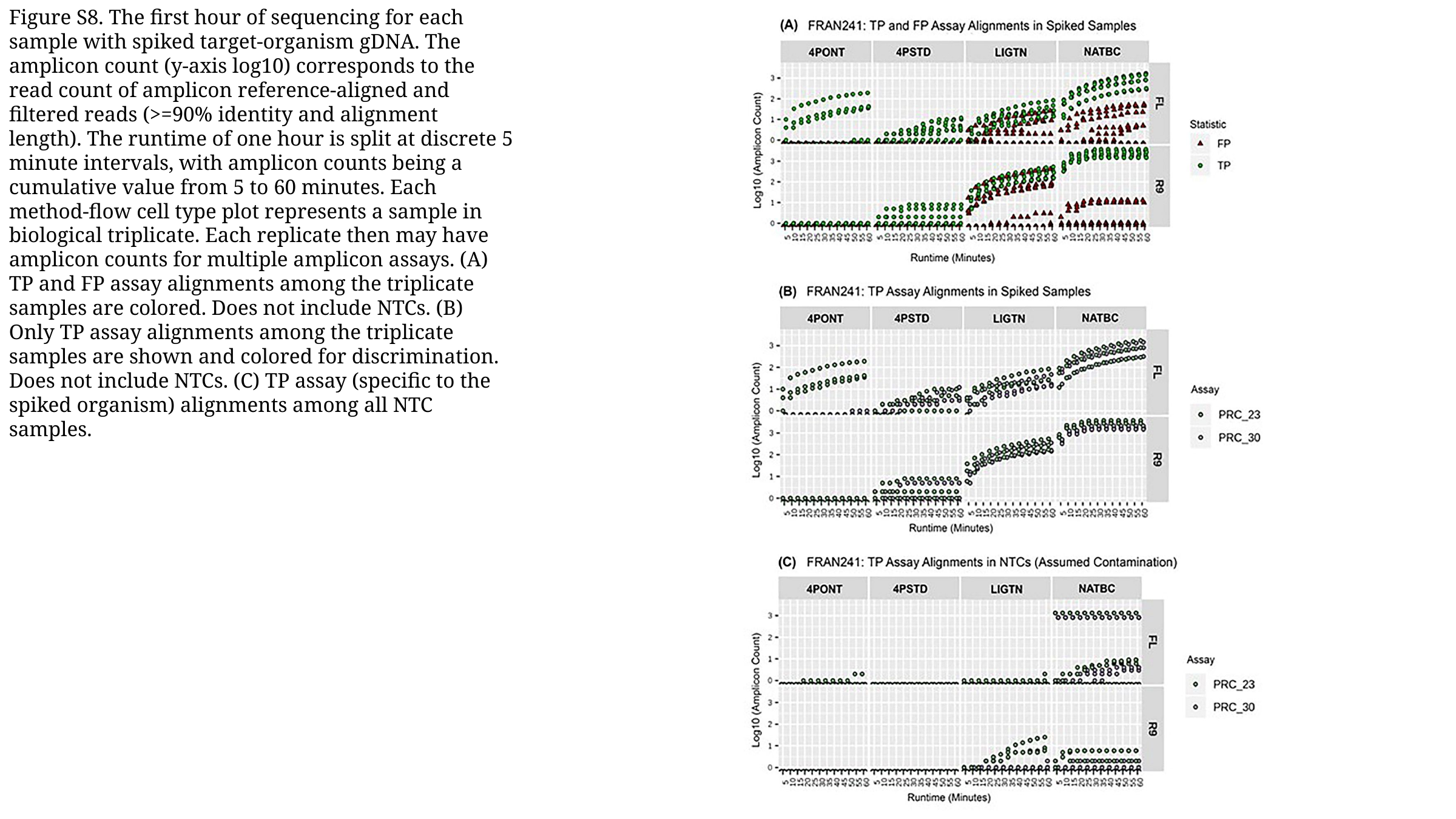

Figure S8. The first hour of sequencing for each sample with spiked target-organism gDNA. The amplicon count (y-axis log10) corresponds to the read count of amplicon reference-aligned and filtered reads (>=90% identity and alignment length). The runtime of one hour is split at discrete 5 minute intervals, with amplicon counts being a cumulative value from 5 to 60 minutes. Each method-flow cell type plot represents a sample in biological triplicate. Each replicate then may have amplicon counts for multiple amplicon assays. (A) TP and FP assay alignments among the triplicate samples are colored. Does not include NTCs. (B) Only TP assay alignments among the triplicate samples are shown and colored for discrimination. Does not include NTCs. (C) TP assay (specific to the spiked organism) alignments among all NTC samples.

## Slide 10
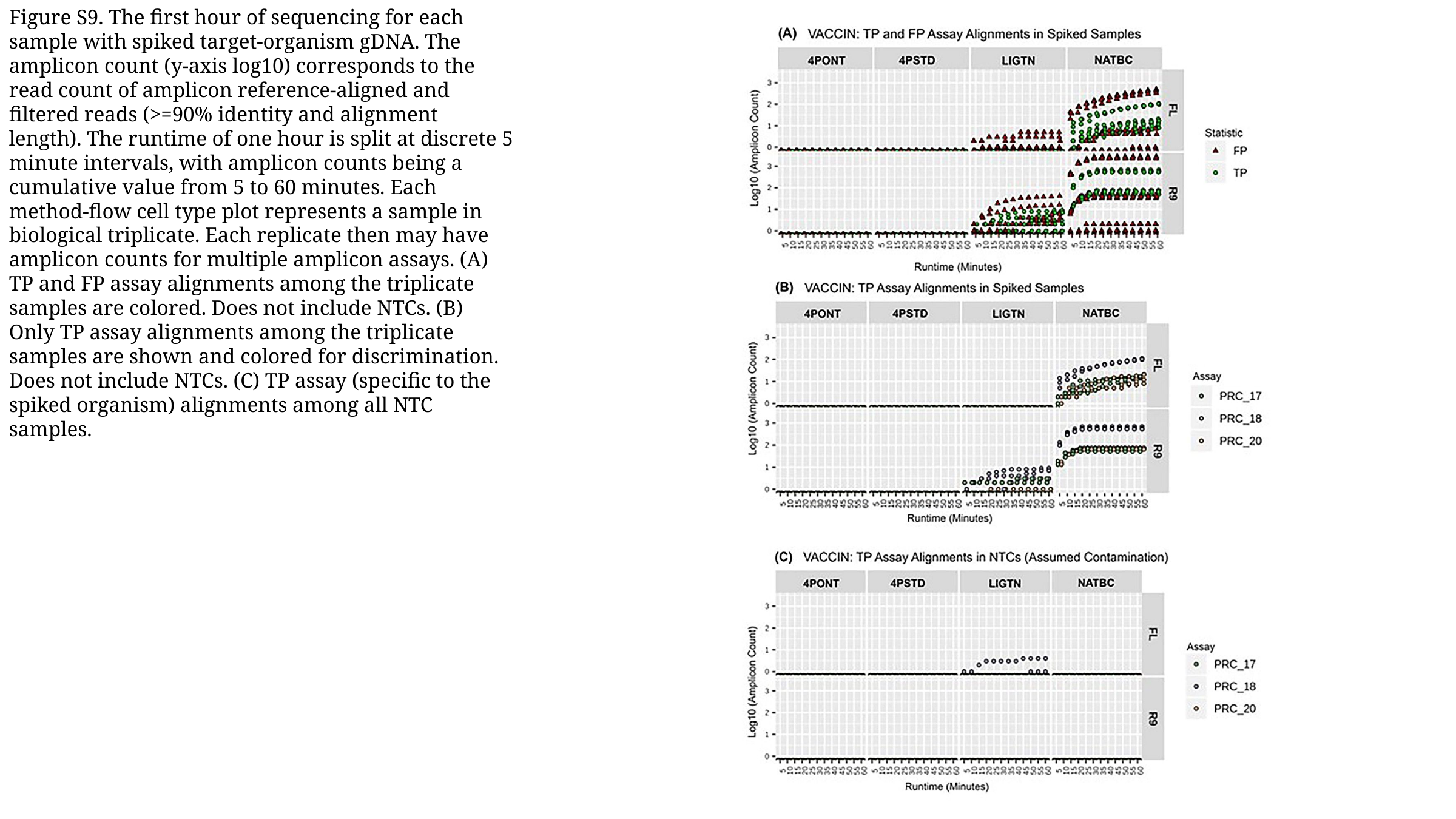

Figure S9. The first hour of sequencing for each sample with spiked target-organism gDNA. The amplicon count (y-axis log10) corresponds to the read count of amplicon reference-aligned and filtered reads (>=90% identity and alignment length). The runtime of one hour is split at discrete 5 minute intervals, with amplicon counts being a cumulative value from 5 to 60 minutes. Each method-flow cell type plot represents a sample in biological triplicate. Each replicate then may have amplicon counts for multiple amplicon assays. (A) TP and FP assay alignments among the triplicate samples are colored. Does not include NTCs. (B) Only TP assay alignments among the triplicate samples are shown and colored for discrimination. Does not include NTCs. (C) TP assay (specific to the spiked organism) alignments among all NTC samples.

## Slide 11
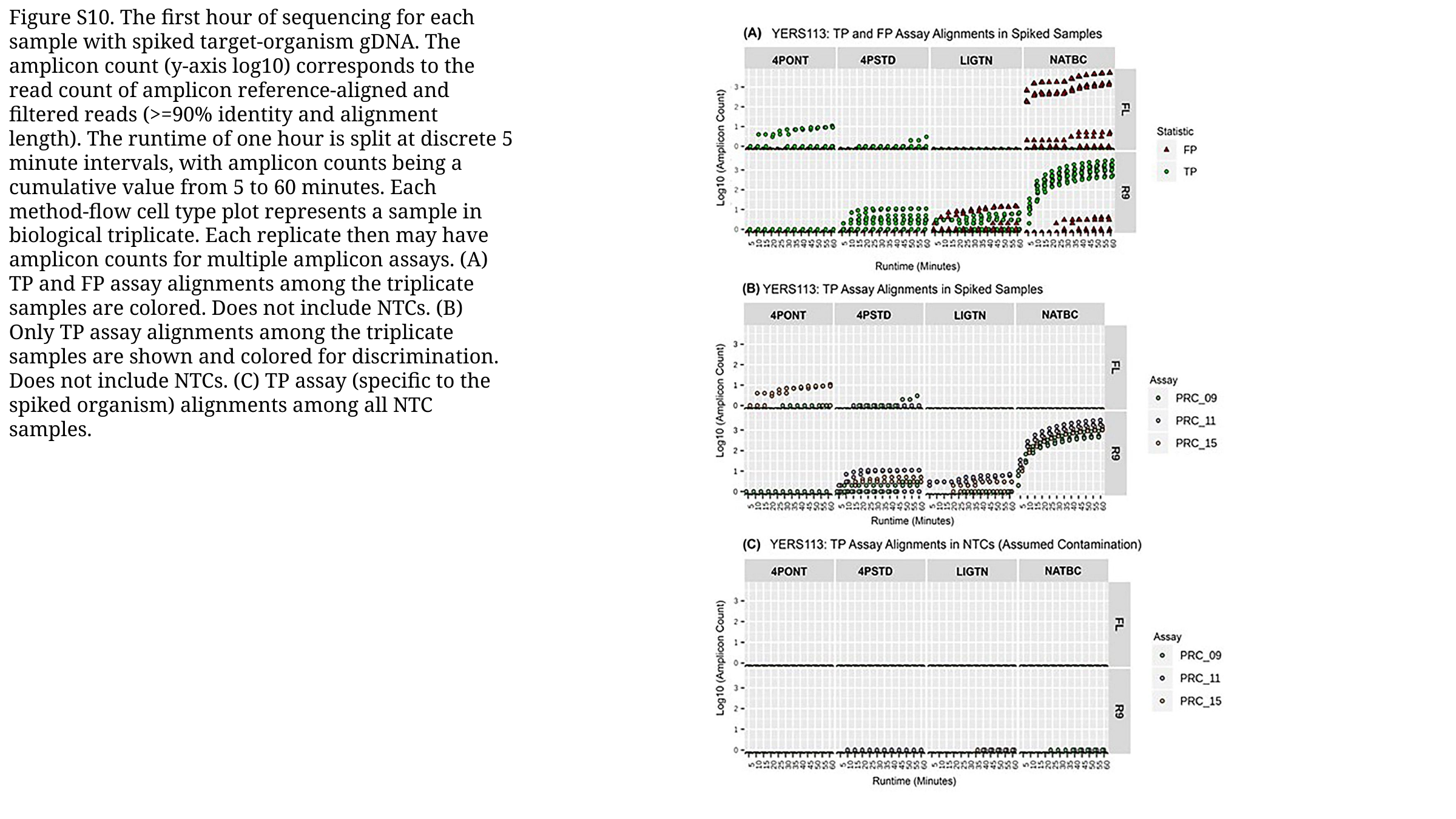

Figure S10. The first hour of sequencing for each sample with spiked target-organism gDNA. The amplicon count (y-axis log10) corresponds to the read count of amplicon reference-aligned and filtered reads (>=90% identity and alignment length). The runtime of one hour is split at discrete 5 minute intervals, with amplicon counts being a cumulative value from 5 to 60 minutes. Each method-flow cell type plot represents a sample in biological triplicate. Each replicate then may have amplicon counts for multiple amplicon assays. (A) TP and FP assay alignments among the triplicate samples are colored. Does not include NTCs. (B) Only TP assay alignments among the triplicate samples are shown and colored for discrimination. Does not include NTCs. (C) TP assay (specific to the spiked organism) alignments among all NTC samples.

## Slide 12
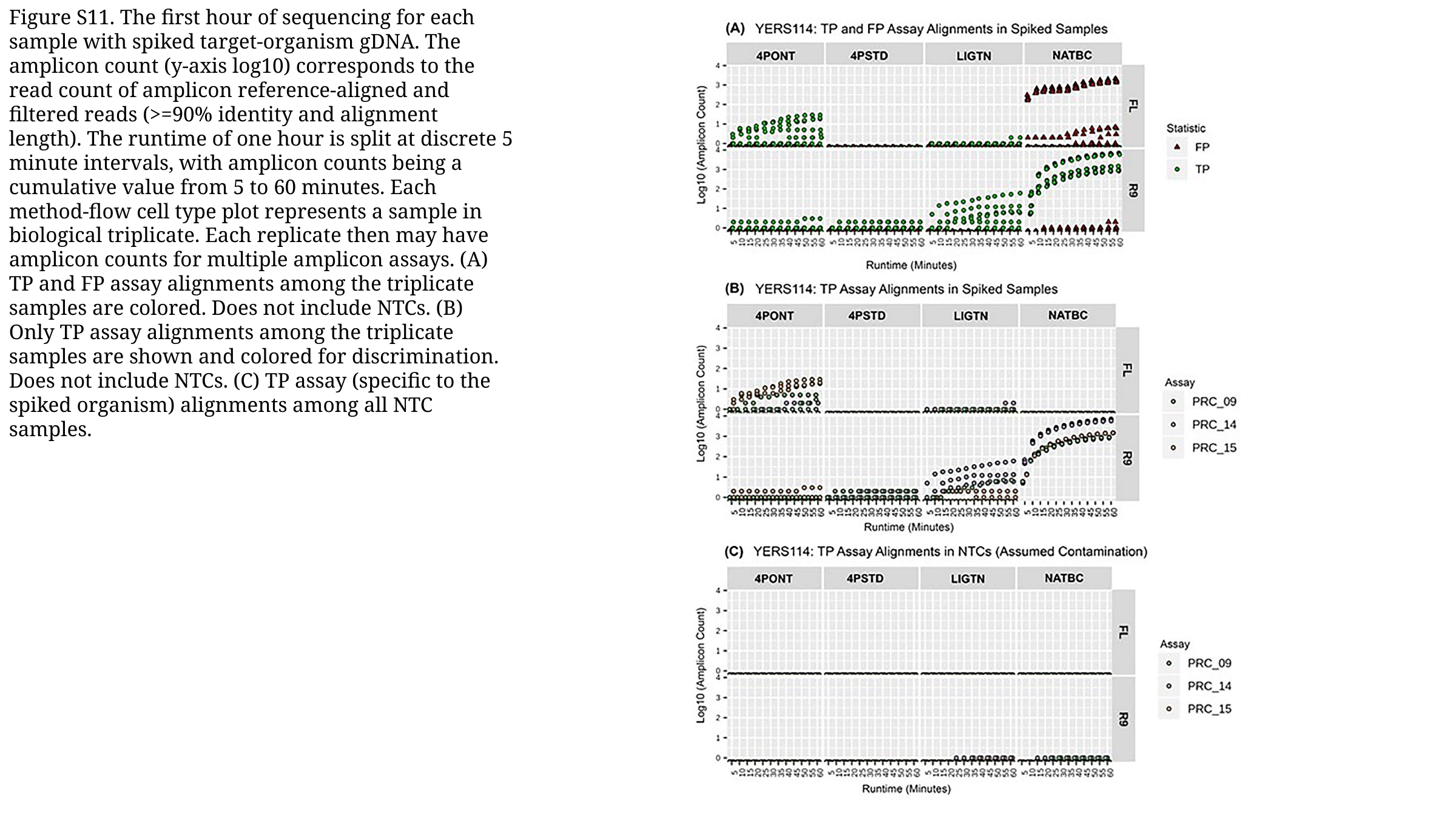

Figure S11. The first hour of sequencing for each sample with spiked target-organism gDNA. The amplicon count (y-axis log10) corresponds to the read count of amplicon reference-aligned and filtered reads (>=90% identity and alignment length). The runtime of one hour is split at discrete 5 minute intervals, with amplicon counts being a cumulative value from 5 to 60 minutes. Each method-flow cell type plot represents a sample in biological triplicate. Each replicate then may have amplicon counts for multiple amplicon assays. (A) TP and FP assay alignments among the triplicate samples are colored. Does not include NTCs. (B) Only TP assay alignments among the triplicate samples are shown and colored for discrimination. Does not include NTCs. (C) TP assay (specific to the spiked organism) alignments among all NTC samples.

## Slide 13
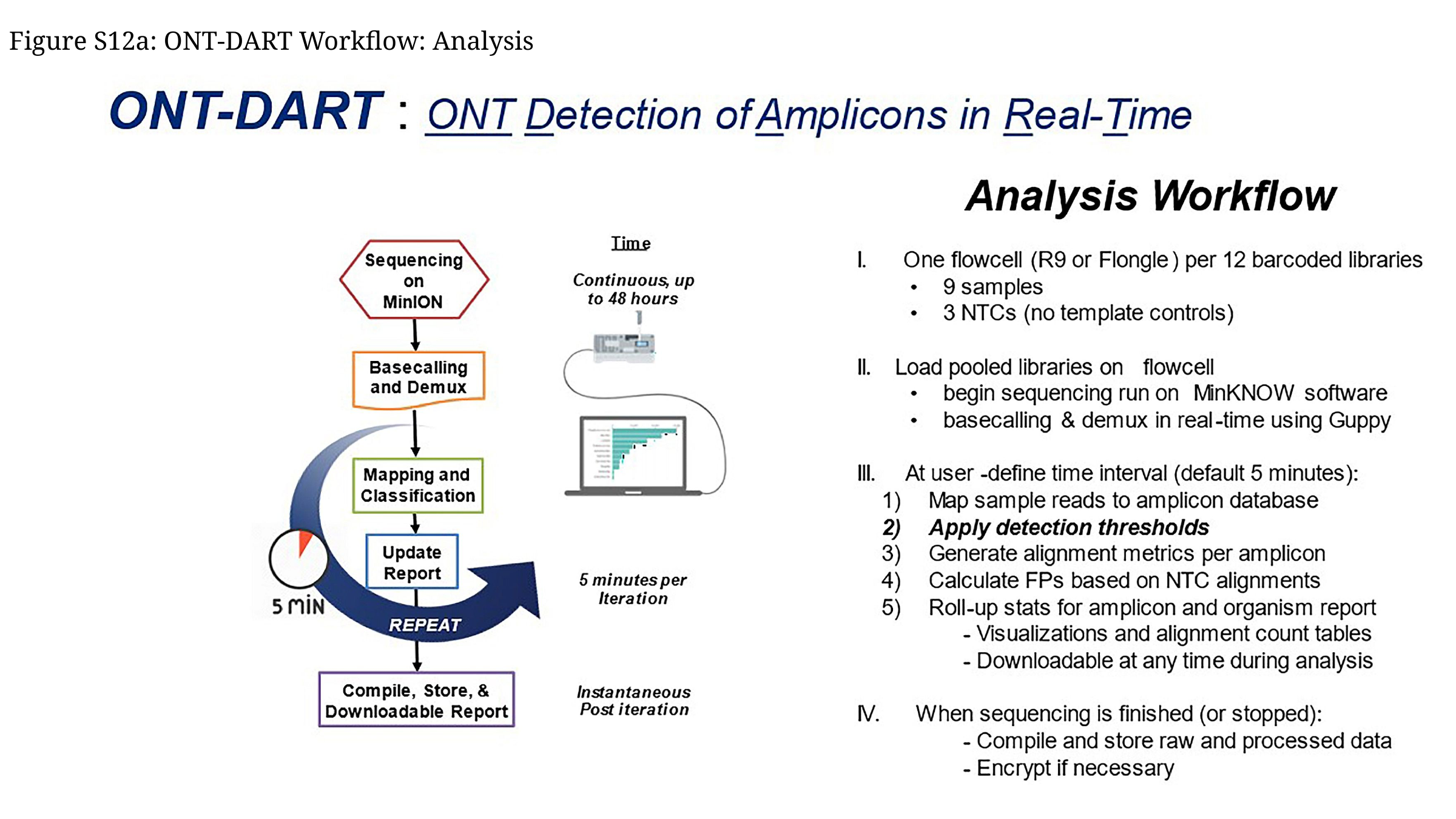

Figure S12a: ONT-DART Workflow: Analysis

## Slide 14
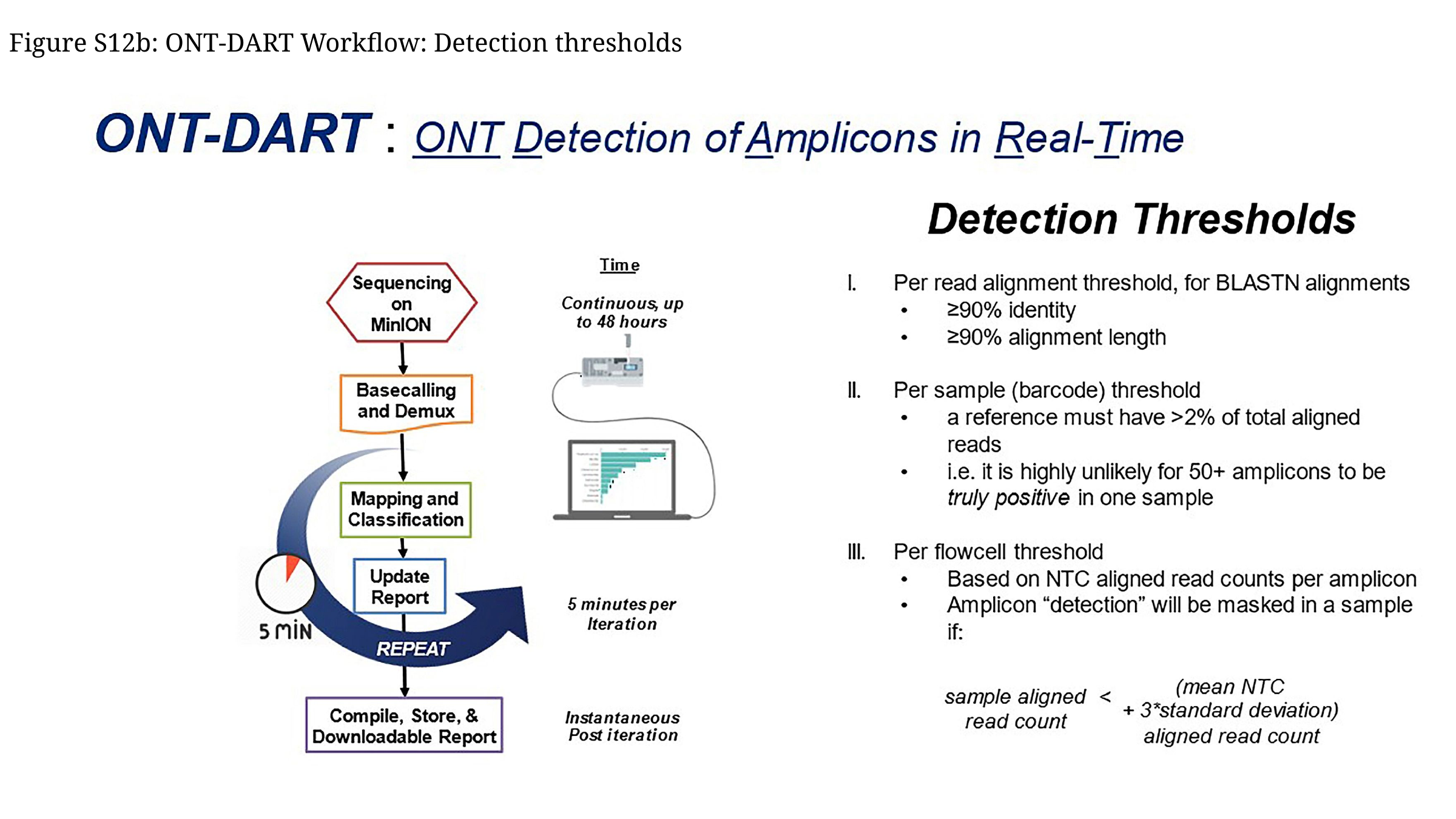

Figure S12b: ONT-DART Workflow: Detection thresholds
